# The Effect of Testosterone Administration and Digit Ratio (2D:4D) on Implicit Preference for Status Goods in Healthy Males

**DOI:** 10.3389/fnbeh.2017.00193

**Published:** 2017-10-16

**Authors:** Yin Wu, Samuele Zilioli, Christoph Eisenegger, Luke Clark, Hong Li

**Affiliations:** ^1^Research Center for Brain Function and Psychological Science, Shenzhen University, Shenzhen, China; ^2^Shenzhen Key Laboratory of Affective and Social Cognitive Science, Shenzhen University, Shenzhen, China; ^3^Behavioural and Clinical Neuroscience Institute, Department of Psychology, University of Cambridge, Cambridge, United Kingdom; ^4^Department of Psychology, Wayne State University, Detroit, MI, United States; ^5^Department of Family Medicine and Public Health Sciences, Wayne State University, Detroit, MI, United States; ^6^Neuropsychopharmacology and Biopsychology Unit, Department of Basic Psychological Research and Research Methods, Faculty of Psychology, University of Vienna, Vienna, Austria; ^7^Centre for Gambling Research at UBC, Department of Psychology, University of British Columbia, Vancouver, BC, Canada; ^8^Center for Language and Brain, Shenzhen Institute of Neuroscience, Shenzhen, China

**Keywords:** steroid hormones, social status, conspicuous consumption, implicit association test, prenatal priming

## Abstract

Testosterone has been linked to social status seeking in humans. The present study investigated the effects of testosterone administration on implicit and explicit preferences for status goods in healthy male participants (*n* = 64), using a double-blind, placebo-controlled, between-subjects design. We also investigated the interactive effect between second-to-fourth digit ratio (2D:4D; i.e., a proximal index of prenatal testosterone) and testosterone treatment on status preferences. Results showed that testosterone administration has no discernable influence on self-reported willingness-to-pay (i.e., the explicit measure) or implicit attitudes towards status goods. Individuals with lower 2D:4D (i.e., more masculine) had more positive attitudes for high-status goods on an Implicit Association Task, and this association was abolished with testosterone administration. These data suggest interactive effects of acute testosterone administration and prenatal testosterone exposure on human social status seeking, and highlight the utility of implicit methods for measuring status-related behavior.

## Introduction

Testosterone, a steroid hormone produced primarily by the gonads, is implicated in dominant behaviors and decision-making process. For instance, lower second-to-fourth digit ratio (2D:4D; a proximal index of high exposure to prenatal testosterone in the womb) is associated with a higher number of correct answers in the Cognitive Reflection Test (CRT; Bosch-Domènech et al., 2014; but see Nave et al., 2017), a task measuring the tendency to override an intuitive response that is incorrect. Recent research suggests that the role of testosterone in human social interaction is best understood in terms of the search for, and maintenance of, social status (Eisenegger et al., 2011). In the Ultimatum Game (UG), the proposer faces the threat of rejection if he or she makes an unfair offer. By making a fair offer, the proposer can prevent being turned down, and the rejection rate is usually high for unfair offers (Güth et al., 1982). Testosterone increases the concern for status in the UG such that the proposers perceive a rejection of their offers as more aversive, leading them to make fairer offers (Eisenegger et al., 2010).

Possessions and goods contribute to defining the self and become an extension of one’s identity. Individuals can acquire and signal their status within social hierarchies by purchasing and displaying luxury goods, a phenomenon termed “conspicuous consumption” (Veblen, 1899; Sivanathan and Pettit, 2010). Pervious research has demonstrated a link between testosterone and consumer behavior. For example, individuals with lower 2D:4D (i.e., more masculinized) were more responsive to the status-related consumption experience such that they were more interested in luxury goods after being primed by mate attraction goals or status display goals (Cornelissen and Palacios-Fenech, 2016). Lower 2D:4D (i.e., more masculinized) was also associated with greater desire to offer erotic gifts to a romantic partner among men with high mating confidence (Nepomuceno et al., 2016a). In one study, salivary testosterone levels increased after driving an expensive sport car (compared to an old station wagon), and this effect was stronger if the experiment took place in a busy downtown area (compared to a semi-deserted highway). Furthermore, the effect of car-induced testosterone increase was enhanced when men’s social status was threatened by the wealth displays of a male confederate in the face of a female moderator (Saad and Vongas, 2009). Taken together, these data suggest a link between testosterone levels and displays of high status. However, whether and how testosterone causally influences attitudes and consumption of status-related goods has not been empirically tested.

The aim of the present study was to investigate the effects of a single dose of testosterone on preference for status goods, in a double-blind, placebo-controlled, between-subjects design. Preference for goods can be measured by the Implicit Association Test (i.e., IAT), which has been employed in recent psychopharmacological studies (De Dreu et al., 2011; Terbeck et al., 2012). The IAT is a reliable technique to assess implicit social evaluation, and has been used extensively in the study of attitudes (e.g., racial bias and stereotype; Greenwald et al., 1998). The IAT has also been utilized in the consumer research such that IAT-measured attitudes could predict brand preference, usage and recognition (Maison et al., 2004). In the current version of the IAT, participants categorized positive words and high-status goods with one key, and negative words and low-status goods with another key. In a different task block, the pairings were reversed such that positive words and low-status goods were categorized together. Participants who hold more positive attitudes towards high-status goods (and/or negative attitudes towards low-status goods) should respond faster in the first block compared to the second block. We hypothesized that this difference would be enhanced following testosterone administration. We further tested whether these effects of testosterone were moderated by second-digit-to-fourth digit ratio (2D:4D; van Honk et al., 2011; Carré et al., 2015), a putative indicator of prenatal testosterone exposure obtained by scanning participants’ right hands, which plays a large role in brain organization and gendered behavior. Lastly, we measured participants’ explicit evaluations of the status goods by obtaining willingness-to-pay ratings in a standard consumer psychology procedure (Rucker and Galinsky, 2008).

## Materials and Methods

### Participants

Sixty-four healthy males (mean age = 22.6 years, *SD* = 1.7; age range = 20–27) were recruited through university advertisements. All participants were screened during a telephone interview to exclude individuals taking psychotropic medications, or having any psychiatric or neurological disorders. We only recruited males, as the dosing and pharmacokinetics associated with single dose Androgel administration are only established for men (Eisenegger et al., 2013). Participants were instructed to abstain from alcohol, caffeine intake and smoking for 24 h before the testing session. Each participant received a single dose of Androgel or placebo gel in a double blind, placebo-controlled, between-subjects design. This study was carried out in accordance with Declaration of Helsinki and was approved by Shenzhen University Medical Research Ethics Committee. Written informed consent was obtained from all participants. Participants were paid 200 Chinese Yuan (~$30) as their reimbursement.

### Testosterone Administration

All sessions started at 13:00 and lasted approximately 4 h. Participants in the testosterone group received a single dose of testosterone gel, containing 150 mg testosterone [Androgel^®^]. Participants in the placebo group received colorless hydroalcoholic gel. The gels were applied on the shoulders and upper arms by a male research assistant who was blind to the purpose of the study. Given the 3 h time lag for effects with testosterone gel administration in healthy males (we have corroborated that salivary testosterone levels peaked 3 h after gel administration in an independent sample, not reported here), we began our experimental tasks 3 h post-dosing (Eisenegger et al., 2013). Cognitive testing also involved two further decision-making tasks, not reported here. During the waiting period, participants rested in the laboratory.

### Validation of the Stimulus Set

We validated the experimental stimuli in an independent male sample (*N* = 27). These participants rated the prestige associated with a series of cars (1 = lowest, 9 = highest). As predicted, our high-status cars (*M* = 7.47, *SD* = 1.27; i.e., Porsche, BMW, Ferrari, Maserati, Mercedes-Benz) were rated as more prestigious than low-status cars (*M* = 2.65, *SD* = 1.04; i.e., BYD, Cherry, Dongfeng, Geely, Great Wall) on average, *t*_(26)_ = 17.12, *p* < 0.001.

### Implicit Association Test

The IAT (Greenwald et al., 1998) involved two target categories (high-status vs. low-status car stimuli) and two attribute categories (positive vs. negative). The order of congruent and incongruent blocks was randomly assigned. The IAT data were analyzed using the algorithm from Greenwald et al. (1998). The first two trials of each block were excluded due to typically long response latencies. Next, we excluded latencies below 300 ms and above 3000 ms as outliers due to anticipation or inattention. The average error rate was 3.73% (*SD* = 2.91%), ranging between 0% and 12.50%. Response latencies were log-transformed for analysis. The IAT effect was calculated as the difference between response latencies for incongruent blocks (high-status stimuli + negative words, low-status stimuli + positive words) compared to congruent blocks (high-status stimuli + positive words, low-status stimuli + negative words; Greenwald et al., 1998), such that higher scores indicate more positive attitudes for high-status goods and/or more negative attitudes for low-status goods.

### Explicit Valuation Measure

For the explicit measure, we presented participants the same car stimuli, and asked them “How much would you be willing to pay for the product featured?”, with 1 = 10% of the retail price of the item, 2 = 20% of the retail price of the item, and increasing intervals of 10% up to 12 = 120% of the retail price. We calculated a difference score between willingness to pay for high-status vs. low-status goods as the dependent variable, with more positive values representing greater explicit preferences for high status goods.

### Digit Ratio Measurement

Digit ratio was measured from an image scan of the right hand, measuring the length of the index (2D) and ring (4D) fingers from the ventral proximal crease to the tip of the finger using Adobe Photoshop. The scan was performed at the start of each testing session, and each participant provided consent for his fingers to be scanned. Two research assistants, who were blind to the purpose of the experiment, measured the 2D:4D ratios on three occasions, and the mean value was used for analysis. Inter-rater reliability was high, *r* = 0.94, *p* < 0.001.

### Mood Measurement

We used the Positive Affect and Negative Affect Scale (PANAS; Watson et al., 1988) to measure state mood before and after testosterone administration.

### Statistical Analysis

We first compared the IAT and WTP scores between the testosterone and placebo conditions using independent-samples *t* tests. We then looked at the interactive effect between testosterone treatment and 2D:4D ratio on these two dependent variables by using linear regression model.

## Results

Participants did not differ from chance in guessing whether they had received testosterone or placebo in the experiment, *χ*^2^ = 0.016, *df* = 1, *p* > 0.1. On the PANAS mood ratings, testosterone had no effect on positive affect (testosterone group, *M* = −0.23, *SD* = 0.58; placebo group, *M* = −0.17, *SD* = 0.42), *t*_(62)_ = 0.49, *p* = 0.62, or negative affect (testosterone group, *M* = 0.02, *SD* = 0.28; placebo group, *M* = −0.11, *SD* = 0.35), *t*_(62)_ = −1.62, *p* = 0.11.

We first investigated whether the testosterone treatment influenced preferences for status goods. Independent samples *t*-test revealed no significant difference in either IAT scores, *t*_(62)_ = −0.63, *p* = 0.53, or self-reported willingness-to-pay, *t*_(62)_ = −0.47, *p* = 0.64.

**Figure 1:**
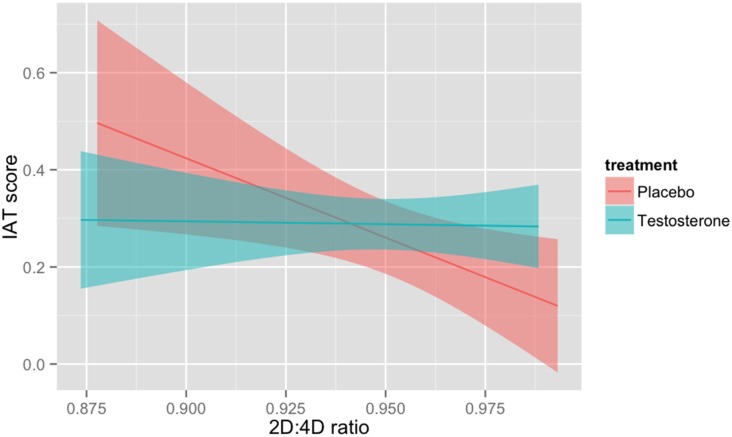
Interactive effect of second-to-fourth digit ratio (2D:4D) and testosterone administration on implicit association test (IAT) score (log-transformed).

Next, in order to investigate the interaction between testosterone administration and 2D:4D ratio, we first regressed IAT scores against testosterone treatment and 2D:4D using linear regression model as Model 1. There was a significant main effect of 2D:4D, *b* = −1.56, *SE* = 0.80, *t* = −1.96, *p* = 0.05. The main effect of testosterone treatment was not significant, *b* = 0.03, *SE* = 0.05, *t* = 0.59, *p* = 0.56. In Model 2, the interactive term between treatment and 2D:4D was entered. The overall linear regression model was significant (*R*^2^ = 0.12, adjusted *R*^2^ = 0.08, *F*_(3,60)_ = 2.85, *p* = 0.04). Adding 2D:4D into the model significantly increased the amount of variance explained, ∆*F*_(1,60)_ = 3.91, *p* = 0.05, ∆*R*^2^ = 0.12. We decomposed the interaction by looking at the relationship between 2D:4D and IAT score in the testosterone and placebo groups separately (see Figure 1). In the placebo group, the association between 2D:4D and IAT score was significant, *b* = −3.26, *SE* = 1.33, *t* = −2.45, *p* = 0.02, suggesting individuals with lower 2D:4D had stronger preference for status-goods. Importantly, this relationship was absent in testosterone administration group, *b* = −0.12, *SE* = 0.85, *t* = −0.14, *p* = 0.89.

To further interpret the significant interaction, we also conducted a simple slope analyses for digit ratio 1 *SD* below the mean and 1 *SD* above the mean (Aiken and West, 1991; Cohen et al., 2013). Testosterone marginally increased IAT scores among individuals scoring relatively high (1 *SD* above the mean) on 2D:4D, *b* = 0.12, *SE* = 0.06, *t* = 1.86, *p* = 0.06, and testosterone had no reliable effect among individuals low (1 *SD* below the mean) on 2D:4D, *b* = −0.06, *SE* = 0.06, *t* = −1.01, *p* = 0.32. As an additional approach to understand the treatment by digit ratio interaction, we created low and high 2D:4D groups by conducting median splits (median split is a valid vobustness check). For individuals in the high 2D:4D group, there was a significant main effect of treatment, *b* = 0.12, *SE* = 0.06, *t* = 1.99, *p* = 0.05. For the low 2D:4D group, the main effect of treatment was not significant, *b* = −0.05, *SE* = 0.07, *t* = −0.75, *p* = 0.46. Thus the median split analyses showed the same pattern as simple slope analyses.

For the explicit measurement, there were no significant main effects of testosterone treatment, *b* = 2.75, *SE* = 14.44, *t* = 0.19, *p* = 0.85, or 2D:4D, *b* = −1.13, *SE* = 11.17, *t* = −0.10, *p* = 0.92, and the interaction term was also not significant, *b* = −2.69, *SE* = 15.19, *t* = −0.18, *p* = 0.86. There was no significant correlation between the explicit measurement and the IAT score, *t*_(62)_ = 1.05, *p* = 0.30.

## Discussion

The present study investigated the effect of testosterone on implicit and explicit preferences for status goods in healthy males. Exogenous testosterone increased IAT scores for status goods among individuals with higher 2D:4D ratios (i.e., less masculine), consistent with past work showing the interaction between testosterone administration and prenatal testosterone exposure in human social interaction (van Honk et al., 2012). The status theory of testosterone predicts that, while in social contexts where status is threatened by perceived provocation (e.g., unfair offers in the UG), this motivation may lead to increased aggression (rejection behavior); in the other case, non-aggressive behavior such as generosity, will be more appropriate for increasing social status (Eisenegger et al., 2011). Using the UG, previous research has found that participants treated with testosterone were more likely to punish the proposer who made unfair offers, and more likely to reward the proposer who made fair offers, consistent with a causal role of testosterone in status-enhancing behaviors dependent on the social context (Dreher et al., 2016). Notably, in the current study, the effect of acute testosterone was driven by individuals with higher 2D:4D ratio (lower prenatal testosterone exposure), consistent with the proposal that the effects of testosterone on social behavior are largely due to metabolism to estradiol, and individuals who are prenatally more primed by estradiol (higher 2D:4D) could metabolize more testosterone into estradiol (van Honk et al., 2012).

The main effect of lower 2D:4D on status preferences on the IAT also corroborates previous research showing that high prenatal testosterone in men predicts courtship-related consumption (i.e., display resources and stastus as to impress women; Nepomuceno et al., 2016b). Lower 2D:4D ratio is associated with more risky choice and more masculine traits such as aggression, dominance and better performance in sports competition (Coates et al., 2009; Sapienza et al., 2009; Apicella et al., 2015). For instance, 2D:4D ratio is significantly associated with risk preferences over lotteries with real monetary incentives (Brañas-Garza et al., in press). Recent research also showed that 2D:4D ratio correlates with social network centrality (Kovářík et al., 2017). In the current study, this association was abolished by testosterone treatment, possibly due to the enhancing effect of testosterone for status-goods among individuals with higher 2D:4D ratio (less masculine).

In the present study, testosterone has no observable effect on self-reported willingness-to-pay. It has been suggested that human social-status seeking often takes various implicit forms rather than being overly explicit, i.e., physical aggression (Eisenegger et al., 2011). The present study used the IAT to measure attitudes for status goods, a technique that is less susceptible to social desirability biases and demand characteristics. This extends recent testosterone research that employs implicit measures such as implicit power motivation, an indirect measure of individual differences in dominance disposition (Stanton and Schultheiss, 2009), in investigating the relationship with dominance behavior. The present data highlight the utility of using IAT as an indirect measure of human status preference.

Some limitations of the study should be noted. First, our experiment tested exclusively male participants since the pharmacokinetic data on testosterone gel is clear in healthy young males (Eisenegger et al., 2013) and social status seeking is more prevalent among males (Eisenegger et al., 2011). Future work would benefit from including both genders in the same design to enable direct comparisons to be tested. Second, we selected the car stimuli only based on the “status” dimension, future work should more precisely control the possible confound of quality (i.e., speed of the cars) or familiarity of the stimuli upon implicit and explicit of evaluation. Third, WTP is a kind of self-report in hypothetical scenario, thus it has no consequence for the decisions the participants made. We encourage future research to utilize incentive-compatible paradigms in measuring preference, e.g., Becker-DeGroot-Marschak auction (Becker et al., 1964).

## Author Contributions

YW, SZ, CE, LC and HL developed the concepts for the study. YW collected the data. YW analyzed the data. All authors contributed to the manuscript and approved the final version of the manuscript for submission.

## Conflict of Interest Statement

LC The Centre for Gambling Research at UBC is supported by funding from the British Columbia Lottery Corporation and the Province of British Columbia. The other authors declare that the research was conducted in the absence of any commercial or financial relationships that could be construed as a potential conflict of interest. The reviewer AME and handling Editor declared their shared affiliation.
